# Correction: Co-expression of DDR2 and IFITM1 promotes breast cancer cell proliferation, migration and invasion and inhibits apoptosis

**DOI:** 10.1007/s00432-024-05636-2

**Published:** 2024-03-28

**Authors:** Chenlu Wu, Jiafei Ying, Mei Dai, Jing Peng, Danhua Zhang

**Affiliations:** 1 Department of Cardiology, The Second Xiangya Hospital, Central South University, Changsha, 410011 Hunan People’s Republic of China; 2 Department of General Surgery, The Second Xiangya Hospital, Central South University, No. 139, Mid Renmin Road, Furong, Changsha, 410011 Hunan People’s Republic of China

**Correction: Journal of Cancer Research and Clinical Oncology (2022) 148:3385–3398** 10.1007/s00432-022-04110-1

In the original version of the article, the Fig. 6I was incorrect. In the Co-immunoprecipitation experiment, the WB blots of the DDR2 in BT20 cells were mistakenly used for twice. Therefore, the WB blots for DDR2 in BT20 cells were unfortunately used for WB blots of IFITM1 in MDA-MB-231 cells in Fig. 6I. The correct figure is provided below.
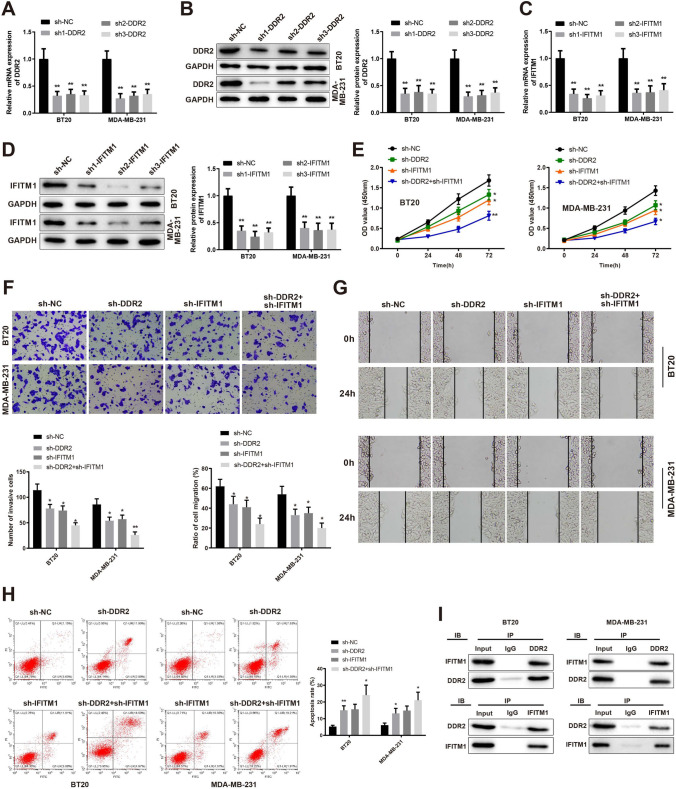


The original article is updated with the correct Fig. 6I.

